# Salicylic Acid Biosynthesis and Metabolism: A Divergent Pathway for Plants and Bacteria

**DOI:** 10.3390/biom11050705

**Published:** 2021-05-09

**Authors:** Awdhesh Kumar Mishra, Kwang-Hyun Baek

**Affiliations:** Department of Biotechnology, Yeungnam University, Gyeongsan 38541, Gyeongbuk, Korea; awadhesh.biotech@gmail.com

**Keywords:** salicylic acid, siderophore, salicylate hydroxylase, isochorismate synthase, salicylate synthase

## Abstract

Salicylic acid (SA) is an active secondary metabolite that occurs in bacteria, fungi, and plants. SA and its derivatives (collectively called salicylates) are synthesized from chorismate (derived from shikimate pathway). SA is considered an important phytohormone that regulates various aspects of plant growth, environmental stress, and defense responses against pathogens. Besides plants, a large number of bacterial species, such as *Pseudomonas*, *Bacillus*, *Azospirillum*, *Salmonella*, *Achromobacter*, *Vibrio*, *Yersinia*, and *Mycobacteria*, have been reported to synthesize salicylates through the NRPS/PKS biosynthetic gene clusters. This bacterial salicylate production is often linked to the biosynthesis of small ferric-ion-chelating molecules, salicyl-derived siderophores (known as catecholate) under iron-limited conditions. Although bacteria possess entirely different biosynthetic pathways from plants, they share one common biosynthetic enzyme, isochorismate synthase, which converts chorismate to isochorismate, a common precursor for synthesizing SA. Additionally, SA in plants and bacteria can undergo several modifications to carry out their specific functions. In this review, we will systematically focus on the plant and bacterial salicylate biosynthesis and its metabolism.

## 1. Introduction

Phenolic compounds contain an aromatic benzene ring with one or more hydroxyl groups produced as secondary metabolites in nature, primarily in plants and some microorganisms [1,2]. They were presumed to be the byproducts of metabolic pathways, and dispensable for important processes common to all organisms [3]. While plants and a few microorganisms (especially bacteria and fungi) produce phenolics, there are variations between and within species [4]. In plants, phenolics play crucial roles in the regulation of different biochemical and physiological processes [5]. One such important phenolic compound is 2-hydroxy benzoic acid, called salicylic acid (SA) [6,7]. There is a wide variation in the basal levels of SA, with up to 100-fold differences among plants of the same family, and significant differences between species [8,9].

Although SA or its related metabolites have long been used as a pain reliever, the active extract of the inner bark of the *Salix alba* was isolated and named ‘salicin’ by a German chemist, Johann Buchner, in 1828 [10]. However, the first report of SA signaling in plants was published in 1987, when a mass spectroscopic analysis of the male flowers of calla lily indicated their role in heat production [11]. Subsequent studies revealed that the reproductive organ of gymnosperms and angiosperms displays a thermogenesis phenomenon due to SA signaling [8,12]. A few years after discovering its role in thermogenesis, SA has emerged as a signaling molecule during pathogen infection [13]. Exogenous SA application into tobacco plant leaves was shown to induce pathogenesis-related proteins and improve resistance to tobacco mosaic virus (TMV) infections [14,15]. Since then, many research groups have demonstrated that the increased levels of SA are associated with the induction of defense genes and systemic acquired resistance in the plant species [16,17]. Afterwards, many studies have established that SA is a key signal molecule in regulating the activation of local and systemic defense responses against infections by pathogens [12,16,18]. With SA being accepted as the ‘sixth’ principal plant hormone in the late 1990s [19], several scientific groups started working on its different physiological roles, barring thermogenesis and plant immunity [16,20,21,22,23]. In a short period of time, SA has become an essential signaling molecule in plants and plays a regulatory role in abiotic stresses, like heat stress and drought, and biotic stresses, such as the systemic acquired resistance mediated defense response against pathogen infection [24,25,26,27,28,29]. In addition, SA’s function also influences plant growth and development by regulating various processes, such as photosynthesis, respiration, vegetative growth, seed germination, flowering, senescence, etc. [30,31,32,33]. Furthermore, acetylsalicylic acid (under the popular trade name, Aspirin) has been an important agent for treating various medical conditions [34]. It has been widely used to manage fever and pain, as well as for the management of cardiovascular diseases [34,35,36] and dermatological conditions such as acne, blisters and pruritus [37].

Chorismate (the end-product of the shikimate pathway) is employed as a primary source for the SA biosynthetic pathway. The shikimate pathway starts with erythrose-4-phosphate and phosphoenolpyruvate, and a series of condensation/redox reactions occur, resulting in the formation of chorismate [38,39]. This exclusively conserved pathway is found in bacteria, plants, and fungi, but not in animals [40]. Chorismate is a central metabolic route for the biosynthesis of aromatic amino acids (L-tryptophan, L-phenylalanine, and L-tyrosine) and various aromatic secondary metabolites (such as alkaloids, flavonoids, lignins, aromatic antibiotics, and SA) [41,42]. Thus, chorismate acts as a common connecting precursor of primary and secondary metabolism [43,44]. The first step in the SA biosynthesis is to convert chorismate to isochorismate by using isochorismate synthase (ICS), or its homologous enzyme, common to both bacteria and plants [20,45]. Seven chorismate-utilizing enzymes exist. Of these, five (ICS, salicylate synthase (SAS), anthranilate synthase, aminodeoxyisochorismate synthase, aminodeoxychorismate synthase) are structural homologues and are collectively known as menaquinone, siderophore, and tryptophan (MST) enzymes [46]. The others, two chorismate-utilizing enzymes (chorismate mutase and chorismate lyase), belong to the non-MST family and are responsible for biosynthesizing phenylalanine, tyrosine amino acid, and ubiquinone. Among the above-mentioned seven enzymes, five are present in both plants and bacteria, while the other two (SAS and chorismate lyase) are exclusively reported in bacteria.

The biosynthesis of SA and its functions in plants have now been completely understood through two breakthrough studies [47,48]. However, knowledge on microbial SA biosynthesis is still scarce, and their functions are yet to be elucidated. In plants, SA functions as a hormone, regulating several physiological processes, such as biotic and abiotic stress responses, seed germination, and flowering [6,24,25]. Meanwhile, in bacteria, SA is mainly associated with salicyl-derived siderophores [49,50], and some of its derivative compounds also act as antibacterial agents, e.g., promysalin [51]. Salicyl-derived siderophores are produced by few plant-growth-promoting rhizobacteria and pathogenic bacteria [52,53]. Considering the practical importance of SA, the objective of this study is to investigate the occurrence of bacterial salicylate, its biosynthetic pathways, and compare with plant SA biosynthesis. Herein, we have illustrated bacterial salicylate production with two crucial examples. Overall, this review confirms that bacterial salicylate is directly correlated with salicylate-derived siderophore biosynthesis.

## 2. Biosynthesis of SA: Overview

SA is usually known as a defense-related plant hormone, regulating various cellular processes. In addition, many bacterial spp. also produce SA as an intermediate compound and they are ultimately incorporated into salicylate-based siderophores. All prokaryotic and eukaryotic organisms require chorismate as a starting precursor for SA biosynthesis. In plants, SA biosynthesis is considered to originate from two pathways: the isochorismate synthase (ICS) pathway (major fraction) and the phenylalanine ammonia-lyase (PAL) pathway [6,47,48]. Both are biosynthetic pathways starting in plastids from chorismate and vary between plant species. In bacteria and fungi, SA or its derivates are produced as natural products [49]. These natural products are secondary metabolites (such as SA, siderophore, aromatic antibiotics, and lignins) and encoded by a set of genes termed as a biosynthetic gene cluster (BGC) [54]. Typically, BGC is found as contiguous clusters on the genome, and their genes encode all enzymes required to synthesize a secondary metabolite. All genes are organized as an operon and expressed jointly [55,56]. There are two common BGC systems: nonribosomal peptide synthetase (NRPS) and polyketide synthases (PKS), which are involved in the majority of secondary metabolite synthesis [57]. The borders of a BGC can be hard to predict because multiple genes of a biosynthetic pathway are often expressed on a single operon and separated by few (30–50) nucleotides. In some scenarios, the genes of a BGC are encoded by multiple operons. However, many computational tools, such as ClusterFinder, antiSMASH, ClustScan, etc., can be used to detect BGCs in the microbial genome [58].

### 2.1. Biosynthesis of SA in Plants

**Figure 1:**
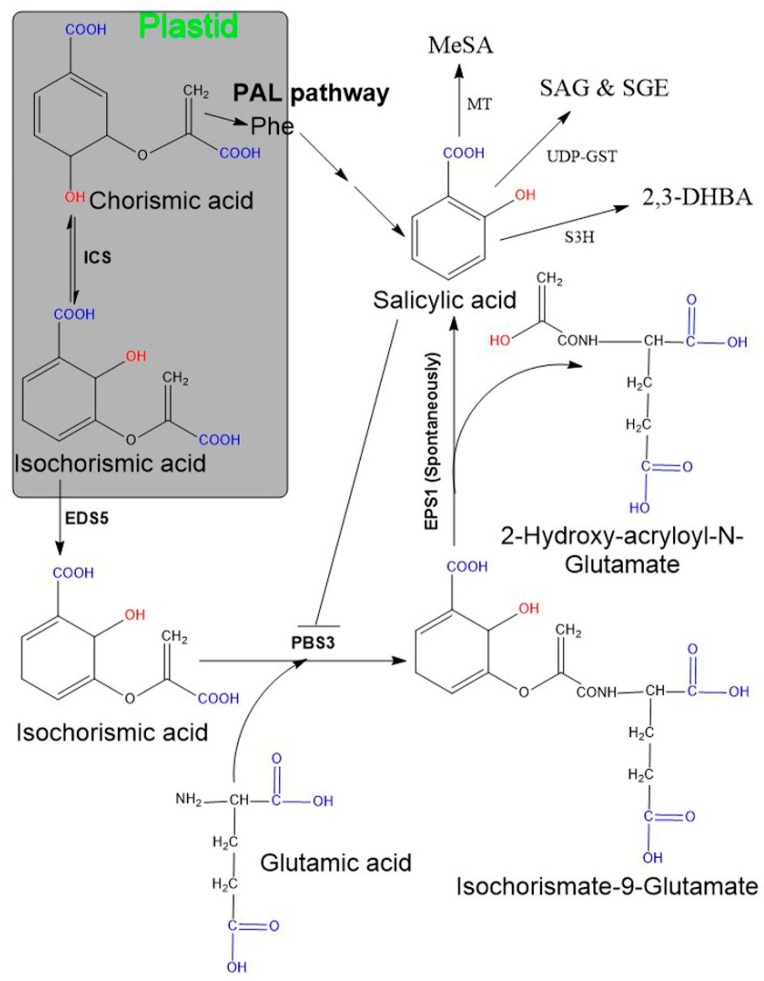
Salicylic acid biosynthesis in plants. Plants possess two pathways (ICS and PAL) and both start from chorisimic acid. In the ICS pathway isochorismate requires two additional genes, named *EDS5* and *PSB3*. The *EDS5*-encoding enzyme exports isochorismate from the plastid to the cytosol. Further, isochorismate attached to glutamic acid with the help of *PBS3* (encoding cytosolic amidotransferase), consequently leads to the formation of an unstable compound, isochorismate-9-glutamate. The non-enzymatic decomposition of isochorismate-9-glutamate then yields salicylate and 2-hydroxy-acryloyl-N-glutamate as final products. The PAL pathway includes the amino acid, phenylalanine as an intermediate compound and comprises multiple sequential enzymatic steps, indicated by several arrows. SA may exist in various modified functional forms, for instance, SAG/SGE, MeSA, 2,3-DHBA. Abbreviation: *EDS5* (*enhanced disease susceptibility 5*), *EPS1* (*enhanced Pseudomonas susceptibility 1*), *PBS3* (*avrPphB susceptible 3*), SAG (SA glucoside), SGE (SA glucose ester), MeSA (methyl salicylate), 2,3-DHBA (2,3-dihydroxybenzoic acid), MT (methyltransferases), UDP-GST (uridine diphosphate glucose-glycosyltransferase) S3H (SA 3-hydroxylase).

Two SA biosynthetic pathways have been elucidated in plants, namely, the ICS and the PAL pathways [6,20,47,48] (Figure 1). Although plants use both biosynthetic pathways simultaneously, ICS is the major pathway contributing to more than 90% of SA synthesis [48,59]. Both pathways originate from a primary metabolic precursor, chorismate. In the ICS pathway, the first step is the conversion of chorismate to its isomer, isochorismate, by the ICS enzyme, and this is found to be a common step in both bacteria and plants [60]. Usually, plants have one or two genes encoding for the ICS enzyme (e.g., Arabidopsis and soybean have two ICS genes, while rice contains only a single ICS gene). Although their activity may vary among plant species, their primary structures are almost conserved [60]. In plants, isochorismate is synthesized in plastids and subsequently transported from plastids to the cytosol with the *enhanced disease susceptibility 5* (*EDS5*) gene. This gene localized on the plastid envelope and encoded for plastidal MATE (multidrug and toxic compound extrusion) transporter enzyme [47,48,61]. Subsequently, ICS is conjugated with L-glutamate and converted to isochorismate-9-glutamate (ICS-Glu) through the cytosolic amidotransferase enzyme. These amidotransferases are encoded by *avrPphB susceptible 3* (*PBS3*; also known as Gretchen Hagen 3.12). Finally, the spontaneous decomposition of ICS-Glu yields SA and 2-hydroxy- acryloyl-N-Glutamate. However, occasionally, an acyltransferase encoded by *enhanced Pseudomonas susceptibility 1* (*EPS1*) may be involved in this final step [22,48]. Moreover, the activity of *PBS3* amidotransferase is inhibited by SA as a negative feedback regulation. Additionally, plants utilize the PAL pathway to synthesize a minor fraction (~10%) of SA and this occurs entirely in the cytosol. Here, the PAL enzyme converts phenylalanine to trans-cinnamic acid (t-CA), and the gene encoding for this enzyme is present in multiple copies in plants [62]. Later, t-CA is converted to SA via two possible intermediates: ortho-coumaric acid and benzaldehyde.

SA in plants exists in two main forms: its active free form and its inactive vacuolar storage form (SA glucoside: (SAG), SA glucose ester: (SGE)). SAG and SGE accumulate in the cell vacuoles in large quantities and can form active, usable forms by hydrolysis [63]. Due to pathogen attack, the total SA (SA + SAG/SGE) level increases enormously and activates the systemic acquired resistance dependent defense pathway. In addition, methylation of SA results in the formation of a volatile form of SA, namely methyl salicylate, which is responsible for increased membrane permeability. Methyl salicylate can serve an important function for plant–insect interactions and systemic acquired resistance signaling [64]. Additionally, hydroxylation of SA results in 2,3-dihydroxybenzoic acid (2,3-DHBA) [65].

### 2.2. Biosynthesis of SA in Bacteria

A large number of different bacterial genera have been reported to synthesize SA, or its related metabolites, especially in plant-growth-promoting rhizobacteria [52]. Among these, the *Pseudomonas* genus is versatile and its presence is well documented. Bacterial SA synthesis seems to be an artifact and usually incorporates SA into SA-derived siderophores (detail discussed in Section 3). In bacteria, secondary metabolite biosynthesis is controlled by a set of two or more locally clustered genes, known as BGCs. They together encode a core biosynthetic enzyme for the backbone of the compound [66]. In addition, BGC also consists of many genes for (1) regulatory enzymes (transcription factors), (2) tailoring enzymes for modifying backbone structure, and (3) enzymes required for transportation and resistance [67]. Diverse structural classes of BGCs exist, such as nonribosomal peptide synthetases (NRPSs) and polyketide synthases (PKSs), bacteriocins, cyclopeptides, terpenes and homoserine lactones, etc. Typically, one BGC accounts for the production of one or several similar bioactive compounds, and they may vary in terms of specificity. Furthermore, the synthesis of some secondary metabolites implicates two or more BGCs.

## 3. SA-Derived (Catecholate) Siderophores in Bacteria

Siderophores are small Fe^3+^-chelating secondary metabolites secreted by bacteria and fungi under low-iron conditions [68]. In addition, some plants also produce siderophores for iron acquisition [69]. Generally, siderophores are produced intracellularly and secreted outside the cell as iron-free (deferri) compounds. After scavenging iron (Fe^3+^ form), the iron-siderophore complex is transported into the cell by the transport system. Based on their structural features and iron-chelating functional groups, siderophores have been classified into three main classes: catecholate (also termed as pheno-catecholate or salicyl-derived siderophore), hydroxamate, and carboxylate [70]. In addition, there are mixed-types (containing more than one of the above-named moieties). In the aforementioned three major classes of siderophores, catecholate siderophores are exclusively produced by bacteria. Usually, bacterial SA is assimilated into the salicyl-derived (termed as salicylates) siderophore backbone. A few mycorrhizal fungi (*Ustilago maydis* and *Phialocephala fortinii*) have also been reported to produce hydroxamate siderophore [71]. Further, the *Pseudomonas* spp. have been documented as the main synthesizer for the catecholate siderophore. More than 100 salicylate siderophores have been reported, and a few are listed in Table 1. Based on their major structural moieties, the catecholate siderophores can be classified into three classes, oxazoline/oxazole, thiazoline/thiazole, and serine-backbone groups. All catecholate siderophores use SA or its hydroxylated derivate, 2,3-dihydroxybenzoic acid (2,3-DHBA), as the common precursor, and its biosynthesis has been well established to involve enzymatic transformations starting from chorismate [72]. Furthermore, several bacteria are capable of producing more than one type of siderophore, such as mycobacterium (produces both types, catecholate and carboxylate).

Siderophore biosynthesis occurs in two ways: the nonribosomal peptide synthetase and polyketide synthases (NRPS/PKSs) pathway, and the NRPS-independent siderophore (NIS) synthetase pathway. Both NRPS/PKSs and NIS biosynthetic enzymes are encoded as BGCs on the microbial genome. Further, each gene is encoded by a specific module and represents a specific enzyme. Bacteria synthesize salicyl-derived siderophores by two methods: either NRPS biosynthetic gene clusters (NRPS BGC) or NRPS/PKS hybrid biosynthetic gene clusters (NRPS/PKS BGC). For example, pyochelin and bacillibactin biosynthesis involves NRPS, while yersiniabactin and mycobactin involve both NRPS/PKS [122]. PKSs are commonly found in fungi, but they are also contained in a few bacteria. Additionally, some bacteria employ other gene clusters along with NRPS/PKS biosynthetic gene clusters [123].

NRPS catalyzes the synthesis of highly diverse natural microbial products, such as antibiotics, toxins, and siderophores [124]. They are found in all three domains of life (bacteria, archaea, and eukarya) and can synthesize a peptide from a variety of standard, non-proteinogenic amino acids, as well as carboxylic and hydroxy acids. Typically, NRPSs are multimodular enzymes consisting of repeated modules (type I NRPS), but nonmodular enzymes (type II NRPS) are also reported [125,126]. In multimodular NRPS, each module contains three domains: adenylation domain, thiolation domain (also termed as peptidyl carrier protein), and condensation domain. Condensation domains of NRPS (which catalyze the amide bond formation) are functionally the most important and suitable target for natural product genomic analysis [125,127]. Nonmodular NRPSs are linear, comprising specialized tailoring enzymes for more diversification. Nonmodular NRPSs commonly combine their substrate with other pathways to generate a final product [126]. All NRPS biosynthetic genes are organized as operons in BGCs, and their regulation takes place at a transcriptional or post-translational level [128,129]. Additionally, salicylate-derived siderophores comprising BGC show significant similarity among the same bacterial genus. For instance, the salicylate-coding gene cluster of *P. fluorescens* was compared with the other three *Pseudomonas* strains and showed a 99% (having 100% query coverage) identity in conserved biosynthetic gene sequences [56].

**Figure 2:**
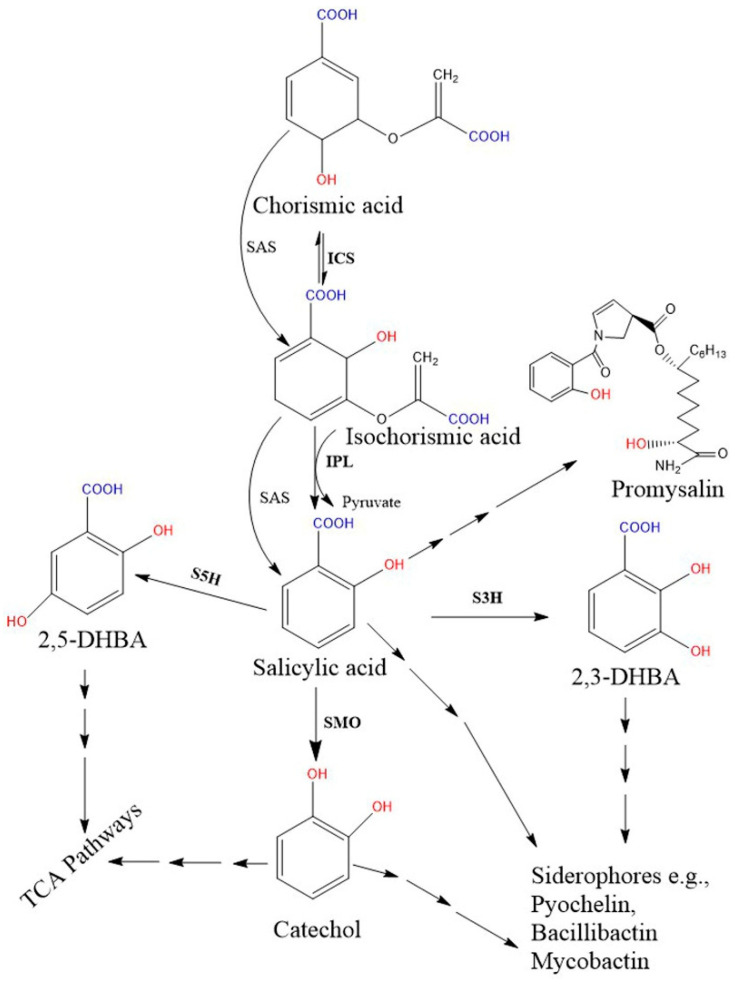
Proposed bacterial pathway for the biosynthesis of SA and their derivative catecholate siderophore. The catecholate siderophore group is only found in bacteria, and their core structures are synthesized from the SA-BGC system. In the *Pseudomonas* spp., SA is synthesized from chorismate via a two-step enzymatic process (*pchA*; ICS and *pchB*; IPL), while the single bifunctional enzyme SAS encoded by *MbtI* (in Mycobacterium) and *Irp9* (in Yersinia) converts chorismate to SA. Further, SMO (also called S1H) converts SA to 1,2-dihydroxybenzene (catechol). Additionally, hydroxylation of SA results in 2,3-DHBA and 2,5 DHBA. Here, salicylate/2, 3-DHBA/catechol can be converted to salicyl-derived siderophores with the help of more than one enzyme. Moreover, catechol and gentisate (2, 5-DHBA) are also metabolized in the TCA pathway. Promysalin, an antibiotic synthesized from SA is also reported in a few *Pseudomonas spp.* [51]. Solid arrows denote single enzymatic steps, while several arrows denote multiple sequential enzymatic steps. All the demonstrated chemical structures are drawn using the ChemDraw 13.0 software (PerkinElmer, Waltham, MA, USA). Abbreviations: ICS, isochorismate synthase; IPL, isochorismate pyruvate lyase; SAS, salicylate synthase; SMO, salicylate1-monooxygenase; S1H, salicylate 1-hydroxylase; S3H, SA 3-hydroxylase; S5H, salicylate 5-hydroxylase; DHBA, dihydroxybenzoic acid; TCA, tricarboxylic acid.

Biosynthetic genes are encoded by a specific module of the NRPSs gene cluster. Two SA biosynthetic routes have been illustrated in bacteria, and the SA is finally incorporated into salicyl-derived siderophores or other metabolites (Figure 2). First, most SA-producing bacterial spp. (e.g., *Pseudomonas* and *Bacillus* spp.) operate an ICS-like biosynthetic pathway. Here, the first step is converting chorismate to its isomer, isochorismate, by the ICS enzyme, while the next step requires another enzyme, isochorismate pyruvate lyase (IPL). IPL converts isochorismate to SA and pyruvate, and is considered a crucial enzyme for bacterial salicylate biosynthesis [45]. So far, no orthologous gene corresponding to bacterial IPL has been identified in plant genomes [7]. Second, some pathogenic bacterial spp. (*Yersinia*, *Vibrio*, *Salmonella,* and *Mycobacterium*) possess a single bifunctional enzyme called salicylate synthase (SAS) that can directly convert chorismate to SA through an isochorismate intermediate. All bacterial biosynthetic genes, *ICS*, *IPL*, and *SAS* are encoded by a specific module of an NRPS BGC. Further, both ICS and SAS enzymes evolutionally originate from a common ancestor and are highly similar to each other [46,113]. The type I NRPS and the type II NRPS siderophores are represented by the vibriobactin siderophore (in *Vibrio)* and mycobactin (in *Mycobacterium)*, respectively.

Salicylate monooxygenase (also known as salicylate 1-hydroxylase) converts salicylate into catechol (1, 2-dihydroxybenzene). This enzyme is also important for salicyl-derived siderophore synthesis and is encoded by a specific module of the NRPS biosynthetic gene clusters [129,130,131]. These enzymes in the *Pseudomonas* species are encoded from the genes of *NahG* in *P. fluorescens* 142 NF and *P. putida* [131,132], *NahW* in *P. stutzeri* AN10 [133], and *NahU* in *P. putida* BS3701 [134].

**Figure 3:**
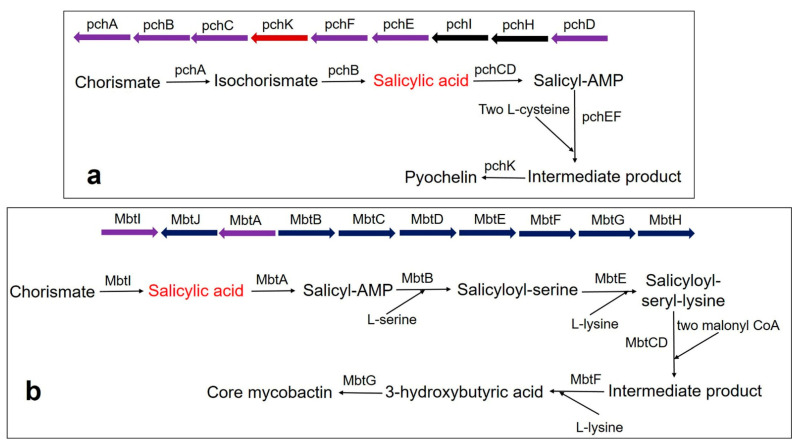
Biosynthesis pathway of catecholate siderophore in bacteria (**a**) Pyochelin biosynthesis in *P. aeruginosa* using BGC comprising the pchDHIEFKCBA operon. The biosynthesis of pyochelin is initiated from chorismate by two salicylic acid synthesizing enzymes *pchA* (ICS) and *pchB* (IPL), encoded by NRPS BGC. Other gene encoding enzymes are *pchC* (type II thioesterase); *pchD* (salicyl-adenylating enzyme); *pchE* and *pchF* (tailoring enzyme that adds L-cysteine and performs cyclization and epimerization); *pchK* (saccharopine reductase); *pchH* and *pchI* (transporter enzyme). (**b**) Core mycobactin scaffold biosynthesis in *M. tuberculosis* by using BGC comprising the MbtA-J operon. Biosynthesis is carried out by a cluster consisting of 10 essential gene encodings for the enzyme (MbtA-MbtJ). Conversion of chorismate to salicylate requires the main enzyme, MbtI. MbtI (salicylate synthase); MbtA (salicyl-adenylating enzyme); MbtB (enzyme for acyl carrier protein); MbtB and MbtE (enzyme for the addition of serine and lysine residues, respectively); MbtC & MbtD (enzyme for the addition of malonyl CoA residues); MbtF (enzyme for the addition of terminal lysine); MbtG (enzyme for N6-hydroxylation of L-lys); MbtJ and MbtH (enzyme encoding unknown functions).

Pyochelin siderophore is made from one molecule of SA and two molecules of cysteine by a thiotemplate mechanism. In *Pseudomonas aeruginosa*, the biosynthesis of pyochelin takes place by the BGC comprising pchDHIEFKCBA operon [135,136,137]. Two enzymes, pchA (ICS) and pchB (IPL), start the synthesis. Chorismate is converted to isochorismate by the pchA and subsequently, the isochorismate is converted to salicylate by pchB. Subsequently, salicylate is activated by pchD (salicyl-adenylating enzyme). In the meantime, enzyme pchC (encoded for type II thioesterase) removes wrongly charged molecules. Afterward, two tailoring enzymes (pchE and pchF) add L-cysteine residues and perform cyclization and epimerization, resulting in the formation of an intermediate product. Finally, the action of pchK (saccharopine reductase) results in pyochelin synthesis (Figure 3a).

Mycobactin biosynthesis is performed by an NRPS/PKS hybrid system in *M. tuberculosis*. The biosynthetic genes are encoded by two BGCs (Mbt-1 and Mbt-2) and include 14 gene encoding enzymes [121]. The Mbt-1 cluster (*mbtA-mbtJ*) consists of 10 essential gene encodings for biosynthetic enzymes (MbtA-MbtJ) involved in core mycobactin scaffold formation (Figure 3b), while Mbt-2 includes four genes encoding for acyltransferase. Mbt-1 consists of salicylate synthase encoded as *Mbt I*, which initiates the synthesis of mycobactin followed by the activation of salicylic acid by the adenylating enzyme, MbtA. Similarly, *V. cholerae* produces the catechol siderophore, vibriobactin, using vibABCDEFH BGC operon. Here, the BGC operon contains SAS encoded by *VibH* [108,138].

In several bacteria, siderophores can act as virulence factors to combat the host immune system [139,140]. Many catecholate-type siderophores are also found in pathogenic bacteria, such as mycobactin (*M. tuberculosis*), vibriobactin (*Vibrio cholera)*, salmochelin (*Salmonella enterica*), petrobactin (*Bacillus anthracis*), and Yersiniabactin (*Yersinia enterocolitica*), and are responsible for their pathogenicity (Table 1 [53,141]. Among all the categories of siderophores, the catecholate-type has stronger iron affinities than transferrin and lactoferrin [142]. These features help it to gain a high degree of pathogenicity in catecholate-producing pathogenic bacteria. Pathogens sequester iron from the host protein using siderophores. To counter this, lipocalin-2 protein is released by the host immune system, which sequesters the catecholate-type siderophores and thus impedes bacterial growth [53,143]. Furthermore, few bacteria, such as *B. anthracis* and *S. enterica*, secrete lipocalin-2 resistant stealth siderophores and evade the host immune system. Hence, siderophores and their biosynthetic enzymes (ICS/IPL/SAS) could act as a suitable target for drugs and could be helpful for medicine development in the future.

## 4. Quantification of Salicylic Acid or Salicylate Siderophore

### 4.1. Quantification of Salicylic Acid in Plant

Quantification of total salicylic acid (both free SA and SA-glucosides) in plant tissues can be done by either using the gas chromatography–mass spectrometry (GC–MS)-based [144,145,146] or the high performance liquid chromatography (HPLC)-based method [147]. For both methods, briefly plant tissues were homogenized in liquid nitrogen and mixed with methanol, followed by centrifugation. Subsequently, the collected supernatant was mixed with extraction solvent such as ethyl acetate–cyclohexane (1:1) (for HPLC-based method) or ethyl acetate (for GC–MS-based method). Afterwards, the sample was analyzed by using a standard amount of SA. GC–MS are highly sensitive approaches that allow more accurate quantification of plant hormones [144].

### 4.2. Quantification of Bacterial Salicylic Acid and Detection of Salicylate Siderophore

#### 4.2.1. Quantification of Bacterial Salicylic Acid

SA synthesis in broth culture can be determined by the previously described method [78,94]. The chosen bacterial strains were grown in Tris-HCl-buffered (100 mM Tris-HCl; pH 7.5) casamino acid media (0.25 g MgSO_4_.7H_2_O, 0.9 g K_2_HPO_4_, 5 g casamino acids in one liter) at 37 °C for 24–36 h. Cells were centrifuged at 3500 revs/min for 15 min and the pH of the culture supernatants were adjusted to 2.0 with the help of 1N HCl. Thereafter, SA was extracted by CHCl_3_ (culture supernatant: CHCl_3_; 3:1) by vigorous shaking. For the quantitative study, one volume of 2.5 mM FeCl_3_ was added to the CHCl_3_ phase. Consequently, the purple Fe–SA complex developed in the aqueous phase, and the absorbance of this complex was measured at 520 nm. SA dissolved in the same growth medium was taken as standard.

#### 4.2.2. Detection of Salicylate Siderophore

Chrome azurol sulphonate (CAS) agar plates were used for regular detection of siderophores [148,149]. However, this assay does not indicate the type of siderophore, and the alternative Arnow’s test was required to be performed for salicylate-derived siderophores [49,150]. This test is based on the reaction between catechol and nitrite-molybdate reagent. The reagent was prepared by dissolving 10 g NaNO_2_ and 10 g Na_2_MoO_4_.2H_2_O in 100 mL of water. Reactants produce a yellow color in an acidic solution, but their color turns to an intense orange-red in alkaline conditions due to the presence of catecholate-type siderophores. Accordingly, bacterial cultures grown overnight were centrifuged at 3500 revs/min and rinsed with 1X phosphate-buffered saline (pH 7.4). Subsequently, 1.0 mL of culture filtrate was mixed with 1.0 mL of 0.5 N HCl, and 1.0 mL of nitrite-molybdate reagent. Then 1.0 mL of 1N NaOH was added and incubated at room temperature for ~5 min, resulting in the generation of an intense orange-red color formation. The intensity of the color is directly correlated with the amount of siderophores. The absorbance was measured at 510 nm with blank (uninoculated growth media) and standard (known concentration of 2,3-DHBA dissolved in the growth medium).

## 5. Conclusions and Future Perspectives

SA is a phytohormone and a secondary metabolite occurring in plants and microorganisms, such as bacteria and fungi. For both plants and bacteria, the biosynthetic pathway requires chorismate, which acts as a central branching point between primary and secondary metabolism. This chorismate is converted to isochorismate, a common step for SA biosynthesis in both plants and bacteria. In plants, the *PBS3* amidotransferase is important for SA accumulation, which catalyzes the conjugation between isochorismate and L-glutamate. The bacterial salicylate production is distinct from that of plants and is often related to the biosynthesis of salicyl-derived siderophores under iron-limited conditions, especially in plant growth-promoting rhizosphere bacteria and pathogenic bacteria. In bacterial species, biosynthetic and regulatory enzymes are encoded by the NRPS BGC on the genome. In salicylate-coding gene clusters, SA biosynthesis starts from chorismate through two pathways, either reaction catalyzed by two different enzymes, ICS and IPL, or a single bifunctional enzyme, SAS. A specific module of bacterial NRPS/PKS encodes these enzymes during the siderophore biosynthesis. In addition, NRPS/PKS also possess many genes for post-translational modification, which leads to structure variability. Several rhizospheric and endophytic bacterial species have been reported as salicylate-producing bacteria, with most of them belonging to *Pseudomonas* genera. Salicylate-derived siderophores play a vital role in the pathogenicity of few bacterial species, and their biosynthetic enzyme serves as a prime target for inhibitory drug development. Although remarkable progress has been made for SA biology in plants, there are still many key questions to be addressed about salicylate biosynthesis and the function of SA-derivatives in bacteria.

## Figures and Tables

**Table 1:** Salicylic acid-producing bacteria and their salicylate-derived siderophores.

Bacteria Species	Salicylate-Siderophore	Bacterial Source	NRPS Biosynthetic Genes	References
*Anabaena cylindrical ^#^*	Anachelin	Pond	ICS-IPL	[73,74]
*Acinetobacter baumannii*	Acinetobactin *	Human	ICS-IPL	[75,76]
*Pseudomonas fluorescens* CHA0	Pyochelin	Tobacco rhizosphere	ICS-IPL	[77,78,79]
*P. fluorescens* WCS374	Pyochelin	Potato rhizosphere	ICS-IPL	[80,81]
*P. fluorescens* WCS417	Pyochelin	Wheat rhizosphere	ICS-IPL	[80,82,83]
*Serratia marcescens*	Pyochelin	Cucumber/Tobacco rhizosphere	ICS-IPL	[84,85]
*P. aeruginosa* 7NSK2	Pyochelin	Barley roots	ICS-IPL	[78,86,87,88]
*P. aureofaciens* 63-28	Pyochelin	Cucumber roots	ICS-IPL	[89]
*P. corrugata* 13	Pyochelin	Cucumber roots	ICS-IPL	[89]
*P. fluorescens* Pf4–92	Pseudobactin *	Chickpea rhizosphere	ICS-IPL	[90,91]
*P. fluorescens* PICF3	Pyochelin	Olive root	ICS-IPL	[81]
*P. aeruginosa RsG18* and *P. aeruginosa* RsG27	Pseudobactin *	Rhizosphere soil	ICS-IPL	[92]
*P. cepacia*	Azurechelin *	Human	ICS-IPL	[93]
*P.tremae*	N/A	Leaves of *Salix babylonica*	N/A	[94]
*Paenibacillus larvae*	Bacillibactin	Larvae of honeybees	ICS-IPL	[95]
*Azospirillum iipoferurn D-2*	N/A	Digireria roots	ICS-IPL	[96]
*Bacillus anthracis*	Petrobactin	Sunflower soil	ICS-IPL	[97]
*Marinobacter hydrocarbonoclasticus*	Petrobactin	N/A	ICS-IPL	[98]
*B. pumilus* SF3	Bacillibactin	Sunflower plant	ICS-IPL	[95,99,100]
*B. subtilis*	Bacillibactin	Banana plant		[100]
*Citrobacter*	Enterobactin	Tomato roots	ICS-IPL	[101]
*Klebsiella pneumoniae*	Enterobactin	Tomato leaves	ICS-IPL	[101]
*Photorhabdus luminescens*	Photobactin	Nematode(*Heterorhabditis bacteriophora*)	SAS	[102]
*Amycolatopsis methanolica* 239^T^	Amychelin	Soil	SAS	[103]
*Salmonella enterica serotype* Typhimurium	Salmochelin	Human	SAS	[104,105,106]
*Vibrio cholerae*	Vibriobactin	Human	SAS	[107,108]
*V. vulnificus*	Vulnibactin	Human	SAS	[108,109,110,111]
*Yersinia enterocolitica*	Yersiniabactin *	Human	SAS	[112,113,114]
*Y. pestis*	Yersiniabactin *	Human	SAS	[115]
*Mycobacterium tuberculosis*	Mycobactin	Human	SAS	[116,117,118,119,120,121]

* Stands for mixed-type siderophore containing salicylates siderophore; ^#^ Stands for cyanobacterium; ^T^ Stands for author name (Tang et al.); N/A stands for not available.

## Data Availability

Not applicable.
